# The role of novel Rydberg-valence behaviour in the non-adiabatic dynamics of tertiary aliphatic amines

**DOI:** 10.1039/c5sc03616j

**Published:** 2015-12-09

**Authors:** James O. F. Thompson, Liv B. Klein, Theis I. Sølling, Martin J. Paterson, Dave Townsend

**Affiliations:** a Institute of Photonics & Quantum Sciences , Heriot-Watt University , Edinburgh , EH14 4AS , UK . Email: d.townsend@hw.ac.uk; b Department of Chemistry , University of Copenhagen , Universitetsparken 5 , DK-2100 Copenhagen Ø , Denmark; c Institute of Chemical Sciences , Heriot-Watt University , Edinburgh , EH14 4AS , UK

## Abstract

Time-resolved photoelectron imaging was used to study non-adiabatic relaxation dynamics in *N*,*N*-dimethylisopropylamine, *N*,*N*-dimethylpropylamine and *N*-methylpyrrolidine following excitation at 200 nm. This series of tertiary aliphatic amines are all of similar chemical makeup, but exhibit differences in their structure – being branched, straight-chain and cyclic, respectively. Our experimental investigation, supported by extensive theoretical calculations, provides considerable new insight into the nature of the internal conversion processes that mediate dynamical evolution between electronic states of predominantly Rydberg character in this important class of model photochemical systems. In particular, the angle-resolved data afforded by the imaging approach (something not previously reported for tertiary aliphatic amines) offers novel and highly-detailed mechanistic information about the overall relaxation pathway. Strikingly, both the experimental and theoretical findings suggest that a critical factor driving the non-adiabatic dynamics is the evolution of valence character along an N–C stretching coordinate within a member of the 3p manifold. This is in stark contrast to primary and secondary amines, as well as many other small hetero-atom containing organic species, where evolution of valence character within the 3s state is now a well-established phenomenon implicated in mediating ultrafast non-adiabatic photochemistry.

## Abstract 2

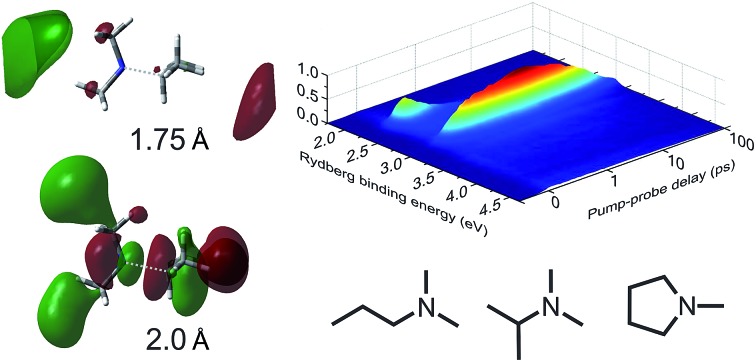
Time-resolved photoelectron imaging was used to study non-adiabatic relaxation dynamics in *N*,*N*-dimethylisopropylamine, *N*,*N*-dimethylpropylamine and *N*-methylpyrrolidine following excitation at 200 nm.

## I. Introduction

Tertiary aliphatic amines (TAAs) provide useful model systems for studying dynamical interactions between electronically excited states in polyatomic molecules following ultraviolet (UV) excitation. Such systems also provide models for nitrogen chromophore centres in a number of naturally occurring species including peptides and neurotransmitters. The electronically excited states of TAAs are all of predominantly Rydberg character.^1^ This offers particular advantages for time-resolved dynamical measurements employing photoelectron detection since spectral features associated with ionization from different electronic states are relatively narrow – and therefore well-resolved – due to a strong propensity for diagonal (*i.e.* Δ*v* = 0) transitions to the cation. A lack of spectral congestion then permits subtle details of the dynamical evolution – for example conformational reorganization – to be readily observed. This is a consequence of Rydberg state energies being extremely sensitive to small changes in the geometry of the ion core, with the observation of such effects in photoelectron data sometimes being dubbed “Rydberg fingerprint spectroscopy”.^2^ This is in contrast to the case of valence state ionization, which typically leads to broad, overlapping ionization features that reflect extended Franck–Condon distributions – often obscuring subtle conformational changes. Rydberg states also possess well-defined electronic orbital angular momentum. This leads to photoelectron angular distributions (PADs) that display pronounced anisotropy. Once again, this may be revealed in an electronic state-specific manner due to the inherently good spectral resolution afforded by Rydberg state ionization. Non-adiabatic coupling between excited states induces mixing of their electronic character and this is often reflected in the temporal evolution of the anisotropy seen in PAD measurements.^3^–^7^ As such, the observation of this evolution using the technique of time-resolved photoelectron imaging (TRPEI) may yield more mechanistic insight into the relaxation dynamics than photoelectron spectra alone. Therefore in principle, utilizing the TRPEI methodology with specifically selected series of TAA systems potentially provides a powerful and generalized “workbench” environment for detailed and systematic investigation of the complex interplay between molecular structure, non-adiabatic dynamics and chemical function following UV excitation – key factors affecting the efficiency (and possibly the evolutionary selection) of many naturally occurring photochemical processes.

As expanded upon further below, many TAAs display clear evidence of “ultrafast” (*i.e.* sub-picosecond) internal conversion between various low-lying Rydberg states. This raises interesting questions relating to the mechanism *via* which such interactions are mediated, since molecular states of purely Rydberg character would not be expected to approach a point of electronic degeneracy along any internal vibrational coordinate until extreme distortions of the molecular framework are sampled. However, the ultrafast nature of the internal conversion strongly implicates conical intersections, rather than slower avoided crossings, as the principle drivers of any non-adiabatic dynamics.^8^ In the case of ammonia and the small (*i.e.* methyl-substituted) primary, secondary and tertiary amines, it is now generally believed that excited states exhibiting predominantly s Rydberg character in the vertical Franck–Condon region evolve significant σ* valence character at relatively modest N–H and/or N–CH_3_ bond extensions.^5^ This may lead to direct dissociation on the initially prepared excited state potential energy surface following UV excitation, but may also facilitate non-adiabatic transitions between different electronic states thereby providing additional relaxation pathways.^9^ More generally, the role played by mixed s-Rydberg/valence states in mediating non-adiabatic relaxation dynamics following UV excitation has been strongly implicated in providing an inherent “photoprotection” in a significant number of biological chromophores.^10^ Consequently, the spectroscopic study of such states has attracted much attention over the last decade.^9^,^11^

In larger TAA systems the key internal coordinates driving non-adiabatic interactions appear to be less well understood. Zewail and co-workers compared ten different amine species using femtosecond time-resolved mass spectrometry (TR-MS) in conjunction with two-photon excitation at ∼8 eV total pump energy.^12^ All TAA systems investigated exhibited similar relaxation dynamics, attributed to ultrafast internal conversion between various high-lying Rydberg states followed by α-cleavage (*i.e.* fission of the first carbon–carbon bond from the amine group) on a timescale of a few picoseconds. Motion along the nitrogen inversion coordinate was implicated as playing an important role in mediating the internal conversion *via* an electronic state with a configuration based upon a 3s ← σ transition.

Subsequently, more recent studies have focused on excitation in TAAs at energies in the region close to 6 eV (an energy now assumed throughout unless otherwise stated). This initially prepares the 3p Rydberg manifold (*i.e.* 3p_*x*_, 3p_*y*_ & 3p_*z*_), with subsequent internal conversion then populating the lower-lying 3s state. These excited states are all built on electronic configurations based principally on promotion of a non-bonding electron from the nitrogen lone pair orbital. Weber and co-workers investigated internal conversion in *N*,*N*-dimethylisopropylamine using a combination of TR-MS and time-resolved photoelectron spectroscopy (TRPES).^13^ Lifetimes described by exponential time-constants of 701 fs and 87.9 ps were reported for the loss of population from the 3p manifold and the 3s state, respectively. In contrast to the earlier Zewail work, fragmentation (primarily α-cleavage) was shown to occur on the cation potential energy surface rather than in the neutral species (although it was noted the excitation energy was ∼2 eV greater in the former instance). It was suggested that fragmentation pathways within the 3s state were too slow to compete effectively with fluorescence decay. However, quantum yield studies in several other amine systems suggest that non-radiative relaxation becomes dominant at excitation wavelengths <220 nm (5.6 eV).^14^–^16^ Of particular note here is a fluorescence lifetime study by Phillips and co-workers,^15^ who reported dual exponential decay in several TAA systems following excitation at energies sufficient to populate the 3s state and 3p manifold simultaneously. This was rationalized by suggesting internal conversion and direct excitation produce different vibrational distributions in the 3s state, leading to the former process having an enhanced propensity for non-radiative decay. This, perhaps surprisingly, implies that vibrational energy is not completely randomized on the nanosecond timescale of the fluorescence experiments. Conceptually related ideas (although operating on much shorter timescales) have also been suggested in more recent results from TR-MS experiments investigating deuteration effects in series of primary and tertiary amines. Here the parent ion lifetime was observed to increase for more massive *N*-substituents.^17^ This is counterintuitive to heuristic “density of states” arguments and was interpreted as an indication that amines, like ketones,^18^–^20^ undergo non-ergodic internal conversion (*i.e.* the nuclear dynamics only sample a reduced phase space before internal conversion).^21^

In trimethylamine, TRPES measurements have revealed sub-picosecond internal conversion within the initially prepared 3p manifold (between the 3p_*z*_ and 3p_*x*,*y*_ states).^22^ Subsequent relaxation from 3p_*x*,*y*_ to the 3s state was then observed on a slightly more extended timescale. Internal conversion within the 3p manifold has also been observed in the larger triethylamine system, along with additional dynamic processes reflecting conformational reorientation of the flexible carbon chains.^23^ Related observations have also previously been reported in several other TAA systems.^24^,^25^ Finally, we note that the UV relaxation dynamics of TAAs containing two amino groups have also been investigated.^26^–^28^ Here interactions between the N atom lone pairs (and associated charge delocalization effects) have been shown to add additional complexity to the dynamics. Such observations are beyond the scope of the present work and are therefore not considered here in detail.

**Fig. 1:**
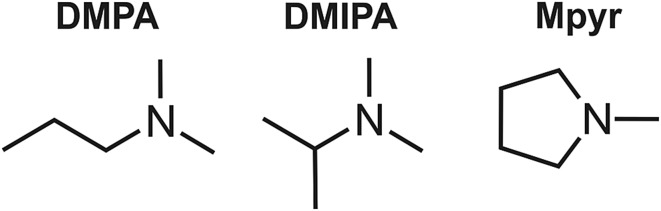
Tertiary aliphatic amines (TAAs) used in the present study: *N*-methylpyrrolidine (Mpyr), *N*,*N*-dimethylpropylamine (DMPA) and *N*,*N*-dimethylisopropylamine (DMIPA).

Here we describe TRPEI studies of three TAA systems following single-photon excitation at 200 nm (6.2 eV). Specifically, these are *N*,*N*-dimethylisopropylamine (DMIPA), *N*,*N*-dimethylpropylamine (DMPA) and *N*-methylpyrrolidine (Mpyr), the structures of which are shown in Fig. 1. All are of similar chemical makeup, but exhibit structural differences (being branched, straight-chain and cyclic, respectively). Our experimental data, supported by extensive theoretical calculations, offer detailed new information regarding the nature of the critical nuclear motions required to access conical intersections that mediate internal conversion in TAA systems, revealing subtle new details of the overall relaxation pathway. To the best of our knowledge, angle-resolved photoelectron measurements have not previously been reported for TAA systems and, critically, it is the PAD data provided by the imaging approach that permits much of this new information to be determined – clearly demonstrating the strength of the highly-differential TRPEI technique for the study of non-adiabatic excited state dynamics.

## II. Experimental methodology

Samples of DMIPA and Mpyr were purchased from Sigma-Aldrich (99% purity). DMPA was synthesized “in-house” by dimethylation of isopropylamine in a standard Eschweiler–Clarke reaction.^29^,^30^ Identity and purity was confirmed by ^1^H-NMR. Prior to commencing photoelectron data collection, preliminary room-temperature UV vapour-phase spectra were obtained for all three molecules using a commercial bench-top spectrophotometer (Camspec M550).

Our TRPEI setup has recently been described in detail elsewhere.^31^ In order to eliminate unwanted cluster formation, samples were placed in a small vessel external to the imaging spectrometer and maintained at 0 °C. Helium (1 bar) was flowed through this liquid sample reservoir and then introduced into the source chamber of a differentially pumped photoelectron spectrometer *via* an Even–Lavie pulsed valve (150 μm nozzle diameter, 1 kHz repetition rate).^32^ After travelling through a skimmer (*∅* = 1.0 mm), the molecular beam passed into the main interaction chamber and was intersected at 90° by co-propagating UV pump and probe pulses. These were derived from the 800 nm fundamental output of a 1 kHz regeneratively amplified Ti : Sapphire laser system (Spectra-Physics, Spitfire Pro/Empower) seeded by a Ti : Sapphire oscillator (Spectra Physics, Tsunami/Millennia Pro). The pump beam (200 nm, ∼0.5 μJ per pulse) and probe beam (267 nm, ∼2.0 μJ per pulse) were provided by generating the fourth and third harmonics of this output, respectively, using thin β-barium borate (BBO) crystals. Temporal pump–probe delay was precisely adjusted using a computer-controlled linear translation stage and these beams were then combined on a thin dichroic mirror and focussed into the spectrometer using a 50 cm focal length UV-enhanced concave aluminium mirror.

Interaction between the UV light pulses and the molecular beam took place between the electrodes of an electrostatic lens set-up optimised for velocity-map imaging.^33^ The resulting photoelectrons were imaged using a 40 mm diameter dual micro-channel plate/P47 phosphor screen detector in conjunction with a CCD camera (640 × 480 pixels). No additional real-time processing of the raw images was performed. To reduce issues with background signals originating from scattering of the 200 nm pump, the entrance and exit windows of the spectrometer were positioned far away from the interaction region by extending side arms off the main vacuum chamber. In addition, small-aperture (2 mm diameter) conical “baffles” were placed inside these arms as close to the interaction region as practically possible. Before commencing photoelectron acquisition, the spectrometer was switched to ion detection mode. This allowed the opening duration and timing conditions of the pulsed valve to be adjusted, ensuring no significant cluster formation was observed in the molecular beam.

Photoelectron data collection repeatedly scanned the pump–probe delay between –500 fs to +1000 fs in 50 fs increments and 19 exponentially increasing steps beyond this point to +100 ps. At each delay position, time-invariant pump-alone and probe-alone images were recorded for subsequent background subtraction. A pump–probe cross correlation of 180 ± 20 fs was obtained directly inside the spectrometer from non-resonant (1 + 1′) multiphoton ionization of nitric oxide. Energy calibration data was obtained from three-photon, non-resonant ionisation of xenon at 267 nm.

## III. Experimental results

### A. UV/VIS spectra

**Fig. 2:**
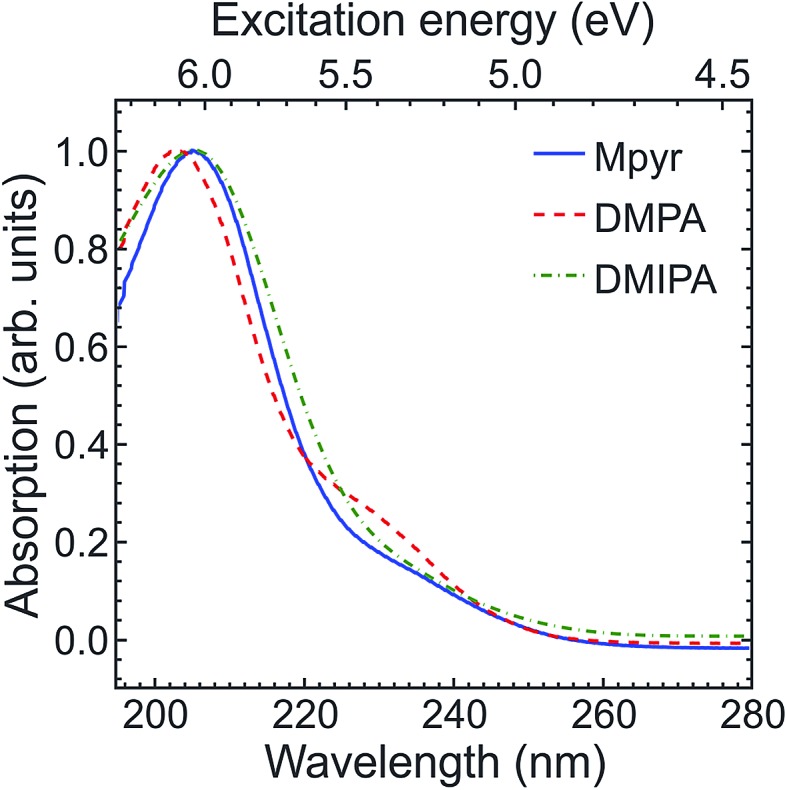
UV vapour-phase absorption spectra of the three TAA systems under study (normalized with respect to the most intense peak).

UV vapour-phase spectra obtained for the three amines under study are displayed in Fig. 2. The spectra are all very similar, showing an intense second absorption band with a maximum in the region 205–210 nm, and a weaker first absorption band appearing as a shoulder at longer wavelengths. As supported by the calculations outlined in Section IV, the first band is attributable to 3s ← n_N_ excitation, and the second band to 3p ← n_N_ (with one member of the 3p manifold excited preferentially over the other two). For the 200 nm pump used in the TRPEI measurements, Fig. 2 confirms that the dominant transition will always be to the 3p manifold rather than the 3s state. Fig. 2 also confirms that a 267 nm probe is well-suited for the TRPEI investigation since it induces no significant absorption in any of the systems under consideration. The data will therefore be free from unwanted “probe–pump” signal evolving to negative time delays.

### B. Time-resolved photoelectron spectra

**Fig. 3:**
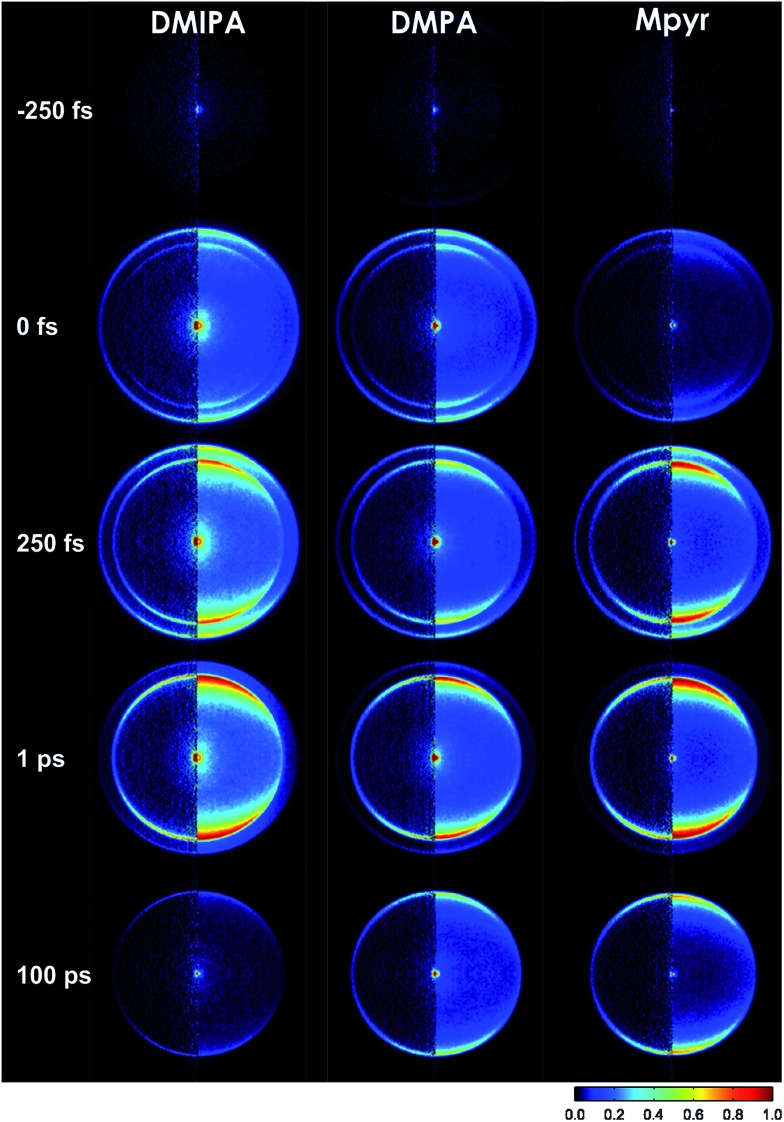
(1 + 1′) photoelectron images obtained for all three TAA systems at selected pump–probe delay times using a 200 nm pump/267 nm probe. Time-invariant pump-alone and probe-alone signals have been subtracted and the images are 4-fold symmetrised. The left half of the images show the processed data obtained following application of the matrix inversion approach described in ref. 31. The (linear) polarization direction of the pump and probe beams is vertical with respect to the figure.

**Fig. 4:**
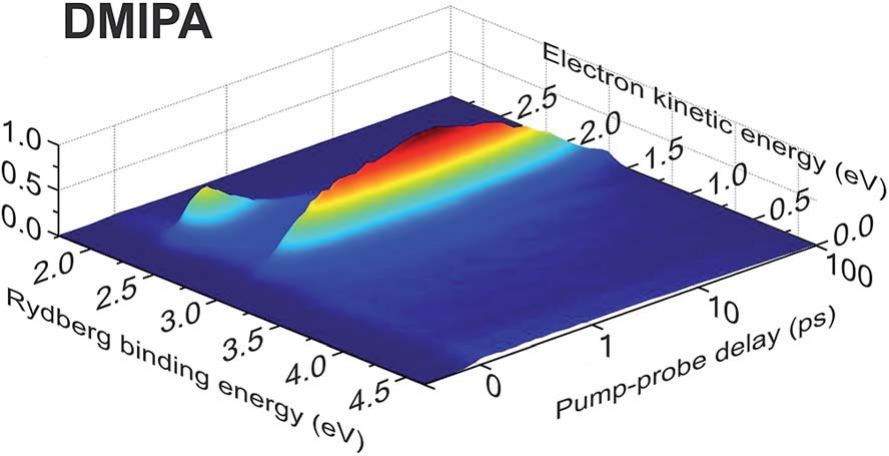
3D time-dependent photoelectron spectrum of DMIPA obtained using a 200 nm pump/267 nm probe. For clear display of the dynamics over all temporal ranges, the time axis is linear to +1 ps and then logarithmic beyond this point. Full width at half maximum Δ*E*/*E* values for the kinetic energies are ∼0.1.

Fig. 3 presents representative photoelectron images resulting from (1 + 1′) ionization at selected pump–probe delays. All systems exhibit sharp ring features with significant anisotropy peaking along the direction of the pump and probe laser polarizations. The left-hand half of each image shows the result following application of a rapid matrix inversion approach that has been described in detail elsewhere.^31^ Application of this inversion, along with appropriate energy calibration, permits time-resolved photoelectron spectra to be generated over the full range of pump–probe delay times for each TAA system. A “3D” example is shown in Fig. 4 for DMIPA. Note here that (i) the time axis is plotted on a mixed linear-logarithmic scale (a feature that will be used throughout) and (ii) for ease of comparison with previous studies,^13^,^34^ the energy axis is plotted in terms of Rydberg state binding energy (*i.e.* photoelectron kinetic energy subtracted from probe photon energy). For completeness, Fig. 4 also includes a photoelectron kinetic energy scale, although Rydberg binding energy will now be used exclusively from this point onwards.

**Fig. 5:**
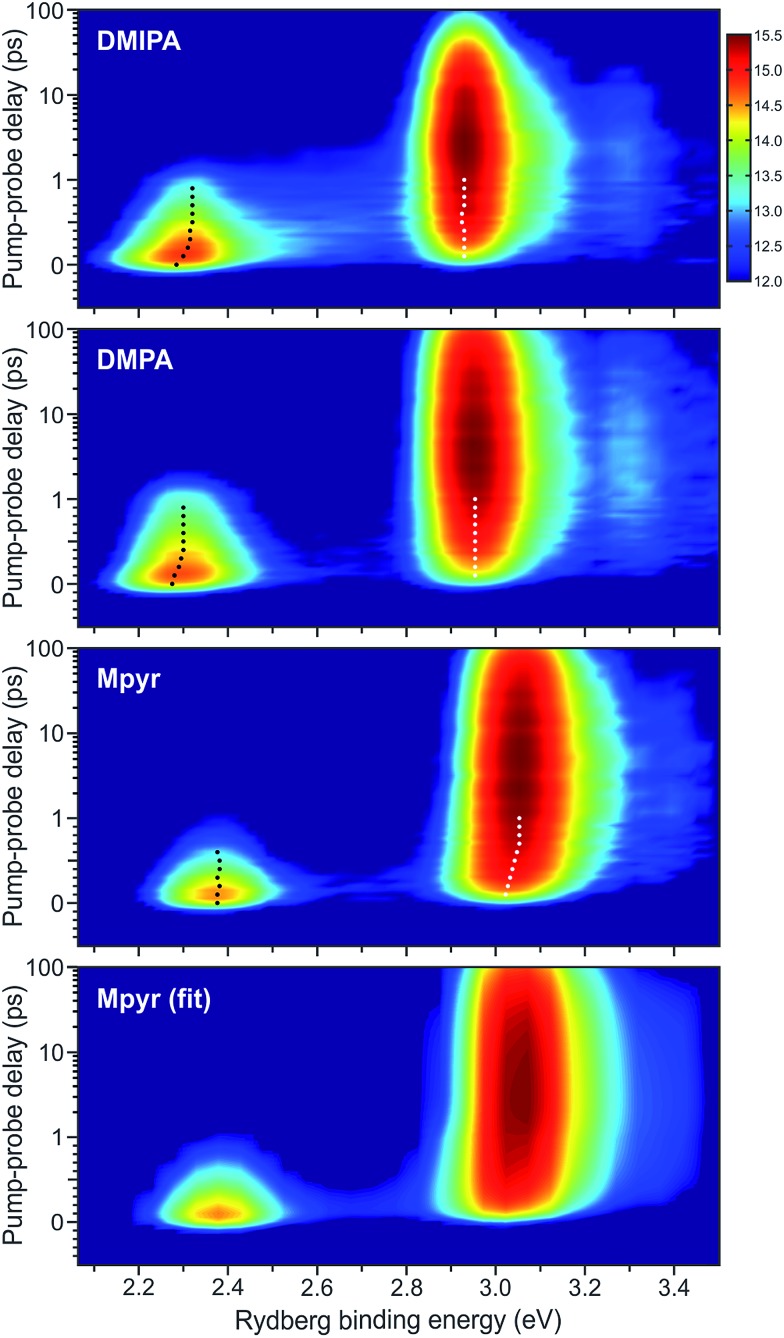
2D time-dependent photoelectron spectra of DMIPA, DMPA and Mpyr obtained using a 200 nm pump/267 nm probe. As in Fig. 4, the time axis is linear to +1 ps and then logarithmic beyond this point. The intensity colour map is presented on a natural logarithmic scale based on the output directly obtained from the imaging CCD camera. Also shown is the fit to the Mpyr data – obtained using the procedure described in the main text. The residual (*i.e.* the fit minus the raw data) displays no observable features on the intensity scale used. Dot overlays denote centre positions of the main peak features obtained from Gaussian fits performed at selected pump–probe delay times.

To make certain features of the data more readily apparent, Fig. 5 presents “2D” time-resolved photoelectron spectra with the intensity plotted on a natural logarithmic scale. Two relatively narrow spectroscopic features are seen in all cases; a rapidly decaying (*i.e.* <1 ps) peak at ∼2.3 eV and a feature at ∼3.0 eV that has a delayed onset from zero pump–probe delay and subsequently decays on an extended (>10 ps) timescale. In DMIPA this longer-time decay is noticeably faster than for DMPA or Mpyr. Based on previous photoelectron studies reported for a number of TAA systems,^13^,^22^–^25^ the lower and higher energy peaks may, respectively, be attributed to ionization from the (initially prepared) 3p manifold and the 3s state (populated subsequently *via* internal conversion). Clear evidence of more subtle dynamical evolution is also evident in Fig. 5 as some of the photoelectron peaks display a distinct shift to higher binding energy with increasing pump–probe delay. In DMIPA and DMPA this shift is associated with the 3p feature whereas for Mpyr it instead appears in the longer lived 3s peak. In all cases there is also an extremely weak feature seen at ∼3.3 eV. This follows the same temporal evolution as the much more intense peak at ∼3.0 eV and is therefore likely due to a small propensity for some non-diagonal (*i.e.*, Δ*v* ≠ 0) ionization from the 3s state involving a high-frequency vibrational mode (presumably a C–H stretch). No other weak spectral features are observed in any other electron energy region (*i.e.* beyond the limited range of Rydberg binding energies shown in Fig. 5).

To analyse the time-dependence of the photoelectron spectra in more detail, global fits were undertaken using a *sequential* Levenberg–Marquardt routine wherein the 2D data *S*(*E*,Δ*t*) are described as:1
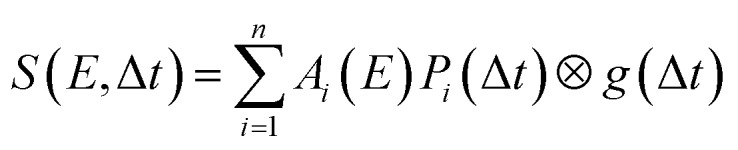
here *g*(Δ*t*) denotes the experimentally determined Gaussian cross-correlation function and *A*_*i*_(*E*) is the decay associated photoelectron spectrum of the *i*^th^ data channel with a time dependent population *P*_*i*_(Δ*t*) defined as follows:

2 *P*_*i*_(Δ*t*) = exp[–Δ*t*/*τ*_*i*_](1 – exp[–Δ*t*/*τ*_*i*–1_])

**Fig. 6:**
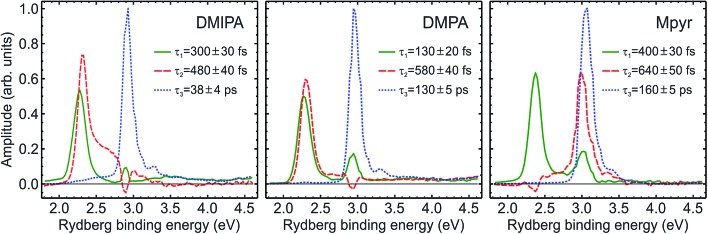
Decay associated spectra (DAS) obtained from a sequential three step global exponential fit to the data presented in Fig. 5. For additional details see the main text. The quoted uncertainties are 1*σ* values and the data is partitioned into 0.025 eV energy bins.

This approach yields a series of decay associated spectra (DAS) plotting the relative amplitude of each fit component as a function of Rydberg binding energy that provides a highly instructive way of visualizing the results from the overall global fit. Using eqn (1), data obtained for each TAA system was fitted with three sequentially decaying functions which we label using their respective time constants *τ*_1–3_. Two of these (*τ*_1_ and *τ*_2_) describe sub-picosecond dynamics while the third (*τ*_3_) models a much longer-lived (38–160 ps) process. DAS plots are presented in Fig. 6. In all cases *τ*_1_ (160–400 fs) exhibits significant amplitude at Rydberg binding energies close to 2.3 eV (*i.e.* ionization from the 3p manifold) although there is also evidence of an additional small amplitude feature at ∼3.0 eV – a consequence of some weak excitation directly populating the 3s state. Since *τ*_1_ is the only function in the overall fit that originates from zero pump–probe delay, such a process must manifest itself in the *τ*_1_ DAS to some extent.

The appearance of the DAS associated with *τ*_2_ (480–640 fs) is striking as the energetic position of the prominent peak produced by the fit is not consistent. In DMPA and DMIPA, this feature appears at slightly higher binding energy than the *τ*_1_ feature at ∼2.3 eV (3p ionization) whereas in Mpyr it is located at slightly lower binding energy than the main *τ*_3_ feature at ∼3.0 eV (3s ionization).

Finally, we note that previously reported TR-MS measurements on DMIPA and DMPA using a 200 nm pump reported exponential decay constants for the parent ion of 619 fs and 739 fs, respectively.^17^ These values are in reasonable agreement with results obtained upon integrating our photoelectron data over the narrow energy region spanning the ionization signal originating from the 3p manifold and then conducting a mono-exponential fit (giving equivalent decay constants of 560 ± 40 fs and 640 ± 40 fs). In DMIPA the lifetime of the 3s state (corresponding to our *τ*_3_ value) has also been shown to depend exponentially on the pump wavelength,^35^ with the time constant of 39.5 ps reported for 202 nm excitation being very close to the 38 ± 4 ps we have obtained here at 200 nm.

### C. Photoelectron angular distributions

**Fig. 7:**
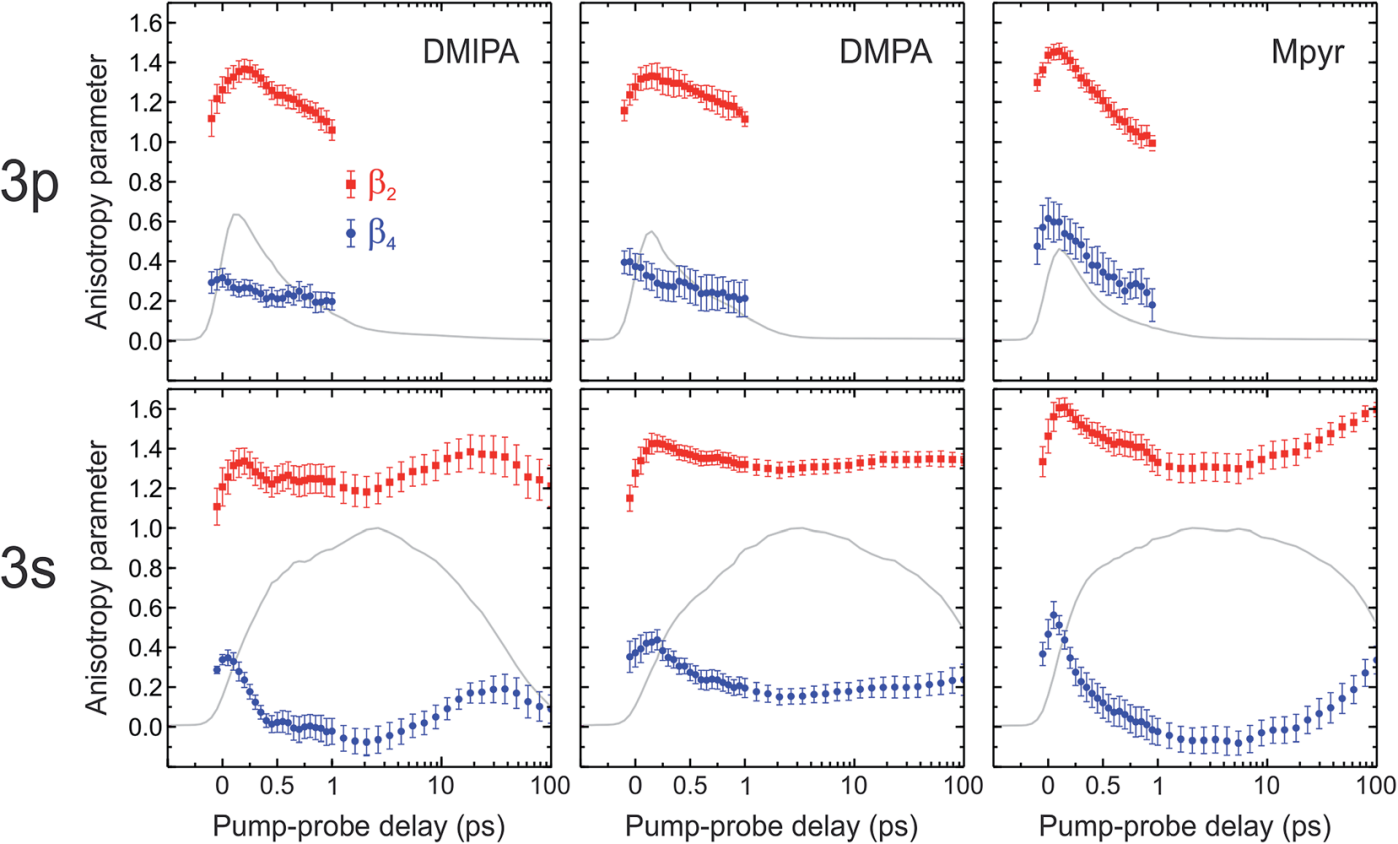
Anisotropy parameters β_2_ and β_4_ as a function of pump–probe delay for 267 nm ionization from the 3p manifold and 3s state. Plots show values averaged over the 0.3 eV energy region spanned by the spectral feature associated with each state. The time axis is linear to +1 ps and then logarithmic to +100 ps. The data was partitioned into 0.025 eV energy bins for the initial anisotropy fits (before averaging) and the error bars denote 1*σ* values. Fits were performed over the angular region 5° ≤ *θ* ≤ 90° to eliminate uncertainties from centre-line noise present in the Abel-inverted images (see Fig. 3). The grey line is the corresponding photoelectron signal within the same energy region.

TRPEI data obtained using (1 + 1′) ionization with parallel linear polarizations yields photoelectron angular distributions (PADs) described by the anisotropy parameters β_2_ and β_4_ as a function of excited state electron binding energy *E* and pump–probe delay time Δ*t*:^36^,^37^
3
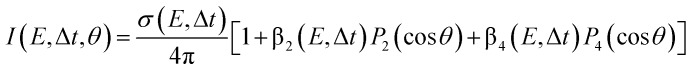
here the *P*_*n*_(cos *θ*) terms are the *n*^th^-order Legendre polynomials, *σ*(*E*,Δ*t*) is the time-dependent electron energy distribution and *θ* = 180° is defined by a vertical line running fully through the images shown in Fig. 3 *via* the centre point. Fig. 7 shows the anisotropy evolution obtained from fits to our PAD data using eqn (3) for the spectral features assigned to ionization from the 3p manifold and the 3s state. The results presented here are averages over the energy regions spanned by each feature. Temporal trends in the data are consistent within each of these regions. Anisotropy parameters presented were obtained using 4-fold symmetrized images, although to ensure no unphysical artefacts were introduced by this process additional analysis was also conducted independently on each quadrant of the un-symmetrized data. Results were consistent in all cases.

The data in Fig. 7 appear broadly similar for all three molecules. For ionization from the initially prepared 3p Rydberg manifold, β_2_ starts by exhibiting relatively large positive amplitude. This then increases slightly, reaching a maximum at Δ*t* ∼ 200 fs, before falling again. For the corresponding β_4_ parameter, no initial increase is apparent but some small decay is observed as Δ*t* increases up to 1 ps. At small pump–probe delays (<1 ps), ionization from the 3s state gives rise to a broadly similar trend for the evolution of both β_2_ and β_4_ to that seen for the 3p manifold. Following their initial evolution, β_2_ and β_4_ then remain constant for ∼10 ps before beginning to slowly rise again. This rise is temporally well-matched to the decay of the associated 3s ionization signal (the grey lines also included in Fig. 7), strongly suggesting that the two observations are related.

## IV. Theory

### A. Excited states

Each TAA system under study was treated to several different theoretical methods to evaluate the energies of the first four singlet excited states (*i.e.* the 3s state and the *x*, *y* and *z* components of the 3p manifold). Initial ground state geometry optimisations were performed using density functional theory (B3LYP)^38^ with an aug-cc-pVTZ basis set. To calculate vertical excited state energies, equation of motion coupled cluster theory including single and double excitations (EOM-CCSD)^39^ was used in conjunction with an aug-cc-pVDZ basis set. In order to investigate the possible need for an improved description of the Rydberg nature of the excited states, calculations using linear response cluster theory including single and double excitations (LR-CCSD) with an aug-cc-pVDZ + (*l*_max_ = 1, *n* = 2–3.5) basis set^40^ were also undertaken. Additional coupled cluster calculations at the non-iterative CCSDR(3) level were performed to investigate the effect of connected triples on the excitation energies.^41^Table 1 shows the various vertical excitation energies and oscillator strengths obtained. The results display an excellent degree of consistency, with both the expanded Rydberg basis and the inclusion of triples exhibiting only very minor influence. The character of the excited states are generally mixed between several Rydberg-type orbitals (as previously reported^42^) but the main component is clearly identifiable and this forms the basis of the stated transition assignments. Standard Gaussian09 orientation conventions are used for the *x*, *y* and *z* axes.^43^ The excitation energies for DMPA and Mpyr are very similar, with those of DMIPA being slightly (∼0.25 eV) lower. In all three systems, one 3p component exhibits somewhat larger oscillator strength than the other two. For Mpyr, this is the lowest-lying member of the series (3p_*y*_) whereas it is the state sitting energetically in the middle of the manifold for DMIPA (3p_*z*_) and DMPA (3p_*y*_). The energy separation between adjacent members of the 3p manifold is relatively small (0.01–0.10 eV) but in some cases this should experimentally resolvable in our TRPEI measurements. Finally, a series of complementary TD-CAM-B3LYP calculations were performed using the aug-cc-pVDZ and aug-cc-pVTZ basis sets. These results are also included in Table 1. In terms of both state energies and oscillator strengths, this density functional theory (DFT) output is in very good agreement with the results obtained from the high-level coupled cluster methods. For the three systems under consideration here, the computationally inexpensive CAM-B3LYP/aug-cc-pVDZ approach therefore provides sufficient accuracy to justify its use in some of the additional investigations outlined below. We anticipate this finding should equally apply to other TAA species and so is potentially also of more general interest.

**Table 1:** Singlet excitation energies and oscillator strengths for all three tertiary amines under study evaluated using several different theoretical approaches. The *x*, *y* and *z* labels for the p states follow the standard Gaussian09 molecular orientation^*a*^

		A		B		C		D		E		F	
		*E*/eV	*f*	*E*/eV	*f*	*E*/eV	*f*	*E*/eV	*f*	*E*/eV	*f*	*E*/eV	*f*
DMIPA	3s ← n_N_	5.16	0.003	5.14	0.003	5.16	—	5.14	—	5.22	0.003	5.24	0.003
	3p_*x*_ ← n_N_	5.69	0.012	5.62	0.012	5.70	—	5.65	—	5.76	0.009	5.77	0.009
	3p_*z*_ ← n_N_	5.81	0.031	5.72	0.021	5.83	—	5.75	—	5.88	0.027	5.88	0.028
	3p_*y*_ ← n_N_	5.85	0.003	5.78	0.006	5.86	—	5.80	—	5.91	0.010	5.90	0.006
DMPA	3s ← n_N_	5.44	0.012	5.41	0.017	—	—	5.42	—	5.47	0.018	5.49	0.017
	3p_*x*_ ← n_N_	5.97	0.011	5.90	0.072	—	—	5.92	—	6.00	0.004	6.00	0.004
	3p_*y*_ ← n_N_	5.99	0.093	5.91	0.100	—	—	5.93	—	6.06	0.101	6.05	0.092
	3p_*z*_ ← n_N_	6.11	0.013	6.07	0.010	—	—	6.07	—	6.10	0.008	6.10	0.008
Mpyr	3s ← n_N_	5.39	0.016	5.35	0.014	5.37	—	5.35	—	5.44	0.014	5.46	0.014
	3p_*y*_ ← n_N_	5.95	0.100	5.84	0.084	5.87	—	5.84	—	6.01	0.080	6.01	0.084
	3p_*z*_ ← n_N_	6.01	0.014	5.94	0.012	5.95	—	5.94	—	6.06	0.034	6.07	0.021
	3p_*x*_ ← n_N_	6.03	0.003	5.94	0.002	5.98	—	5.94	—	6.08	0.001	6.08	0.001

^*a*^A: EOM-CCSD/aug-cc-pVDZ; B: LR-CCSD/aug-cc-pVDZ + (*l*_max_ = 1, *n* = 2–3.5); C: CCSDR(3)/aug-cc-pVDZ; D: CCSDR(3)/aug-cc-pVDZ + (*l*_max_ = 1, *n* = 2–3.5); E: CAM-B3LYP/aug-cc-pVDZ; F: CAM-B3LYP/aug-cc-pVTZ.

Low-lying singlet and triplet state energies for all three systems obtained at the EOM-CCSD/aug-cc-pVDZ level of theory are shown in Table 2. A key point of note here is that the triplet states are only shifted down in energy relative to the equivalent singlet states by ∼0.05–0.15 eV. Table 2 also presents excited state property calculations evaluated for DMIPA at the LR-CCSD level in order to further characterize the excited states. The isotropic invariant of the second moment of the charge distribution relative to the ground state Δ*r*_iso_^2^ has been shown to be an excellent indicator of Rydberg/valence composition^44^,^45^ and we have used this previously to quantify changes in electronic state character during the evolution of associated nuclear dynamics.^5^,^46^ Here we observe that all excited states have large Rydberg character at the S_0_ equilibrium geometry, as indicated by Δ*r*_iso_^2^ = 15 to 19 Å^2^. We have also computed the corresponding isotropic polarizability volumes *ᾱ* for the excited states. Recently we have started exploring the link between *ᾱ* and low-energy (ground state) photoionization cross-sections, revealing a highly significant positive linear correlation that has previously proved instructive in the interpretation of TRPEI data.^5^,^46^ It is therefore interesting to note that *ᾱ* for the 3s state is ∼2–4 times larger than that obtained for the members of the 3p manifold. This observation would seem to fit well with the fact that the 3s ionization signals seen in Fig. 4 are approximately twice as large as those originating from 3p ionization – especially since the strong propensity for diagonal ionization in these TAA systems removes any convoluting Franck–Condon issues.

**Table 2:** Singlet and triplet excitation energies evaluated at the EOM-CCSD/aug-cc-pVDZ level of theory. The S_1–4_ transitions are the same as those presented in Table 1

		T_1_	S_1_	T_2_	S_2_	T_3_	S_3_	T_4_	S_4_
DMIPA	*E*/eV^*a*^	5.09	5.16	5.64 (*x*)	5.69 (*x*)	5.76 (*z*)	5.81 (*z*)	5.83 (*y*)	5.85 (*y*)
	Δ*r*_iso_^2^/Å^2^^*b*^	—	15.43	—	16.03	—	19.38	—	19.32
	*ᾱ*/Å^3^^*b*^	—	221.2	—	51.0	—	135.7	—	59.4
DMPA	*E*/eV^*a*^	5.29	5.43	5.89 (*x*)	5.97 (*x*)	5.93 (*y*)	5.99 (*y*)	6.08 (*z*)	6.11 (*z*)
Mpyr	*E*/eV^*a*^	5.23	5.39	5.84 (*y*)	5.95 (*y*)	5.96 (*z*)	6.01 (*z*)	5.99 (*x*)	6.03 (*x*)

^*a*^EOM-CCSD/aug-cc-pVDZ.

^*b*^LR-CCSD/aug-cc-pVDZ.

All DFT, EOM-CCSD, and CASSCF (see below) calculations were performed with Gaussian09,^43^ while the linear response coupled cluster calculations were undertaken using Dalton2015.^47^,^48^

### B. Conformational considerations

As well as providing a starting point for evaluation of excited state energies, ground state calculations were also used to reveal a number of low-energy conformers for each amine system. Initial conformer searches were performed using Gaussian09 (B3LYP/aug-cc-pVDZ). Following identification of these structures, the geometries were then optimized using second order Møller–Plesset perturbation theory. Separation between the global minimum and the next lowest energy conformer was found to be >90 cm^–1^ in all cases. Multiple ground state conformers are therefore not expected to be present to any significant extent in the jet-cooled molecular beam conditions of the TRPEI experiment and any conformational dynamics observed may be attributed solely to excited state processes.

**Fig. 8:**
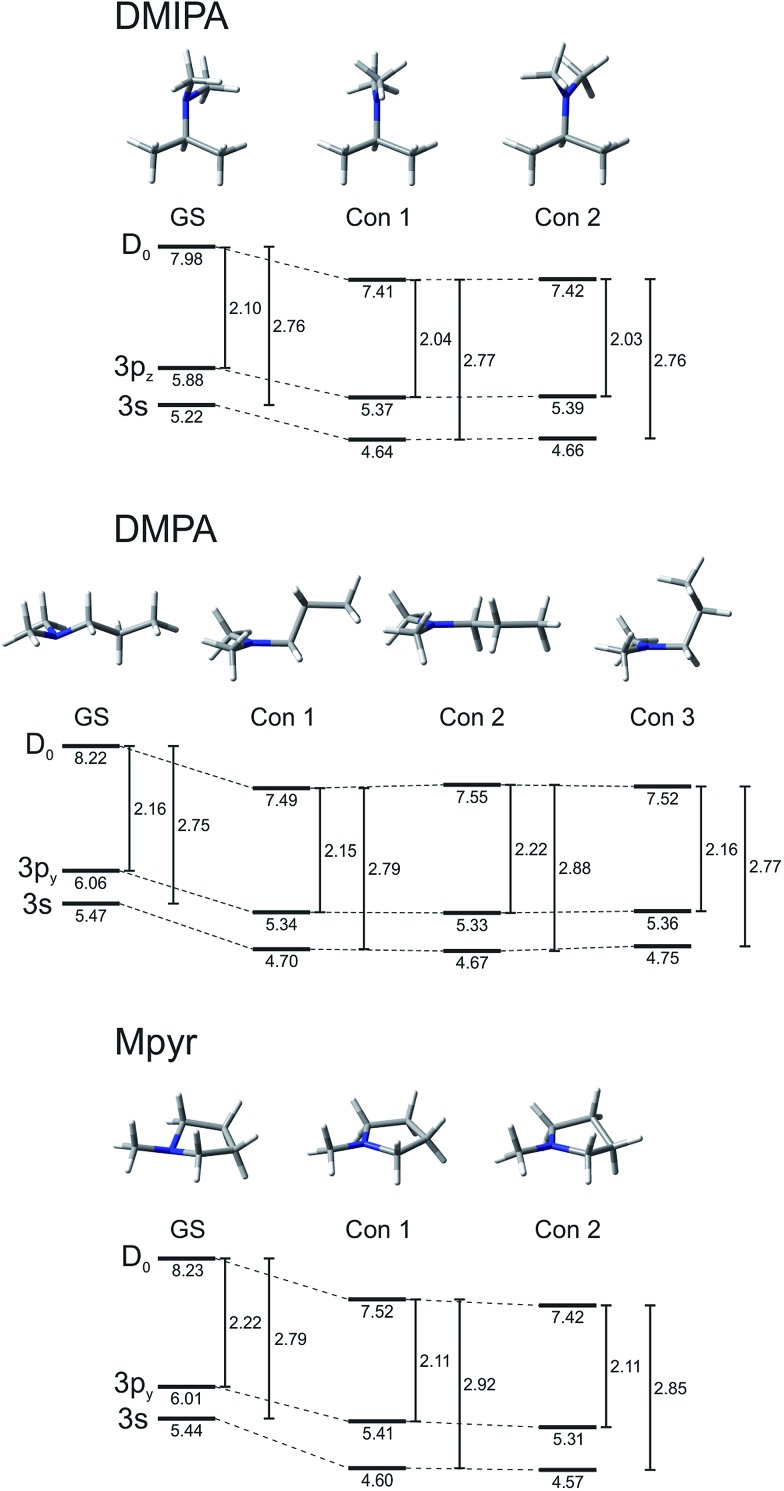
Energy relaxation diagrams for all three TAA systems. State energies at the ground state (GS) geometry and for various relaxed conformers are shown along with the energy gap to the *D*_0_ state of the cation (in eV). For additional details see main text.

Calculations were also performed to investigate conformer structures in the previously identified excited states of all three systems under investigation. Excited state conformers were generated on the ground electronic state of the cation (*D*_0_) using unrestricted DFT (UCAM-UB3LYP) in combination with an aug-cc-pVDZ basis set. This approach was utilised as all excited states identified are of predominantly Rydberg character. The cation geometry may therefore be considered a good approximation to that of the excited states. Values returned from these calculations were subsequently corrected to include the additional energy required to access this part of the potential from the true ground state minima. A similar approach has been previously employed by Weber and co-workers.^23^ Conformer energies, along with the predicted structures, are shown in Fig. 8. Only the 3p state with the highest oscillator strength is shown. The relaxed excited state conformers all exhibit a planar geometry about the N atom. In DMIPA and Mpyr, two structures were identified whereas three were found in DMPA. In all cases the energy gap between the 3s state and the *D*_0_ state of the cation increases slightly (0.02–0.15 eV) as conformational relaxation proceeds from the vertical Franck–Condon geometry. In contrast, the gap between the 3p state and *D*_0_ in DMIPA and Mpyr undergoes a small (0.06–0.11 eV) decrease. In DMPA the equivalent gap remains almost constant or, for one of the conformers, increases slightly. Overall these finding illustrate that conformational rearrangement effects in all three systems should manifest as small (∼0.1 eV), but potentially experimentally resolvable shifts in the photoelectron peak positions associated with ionization from specific Rydberg states.

### C. Dissociative relaxation

**Fig. 9:**
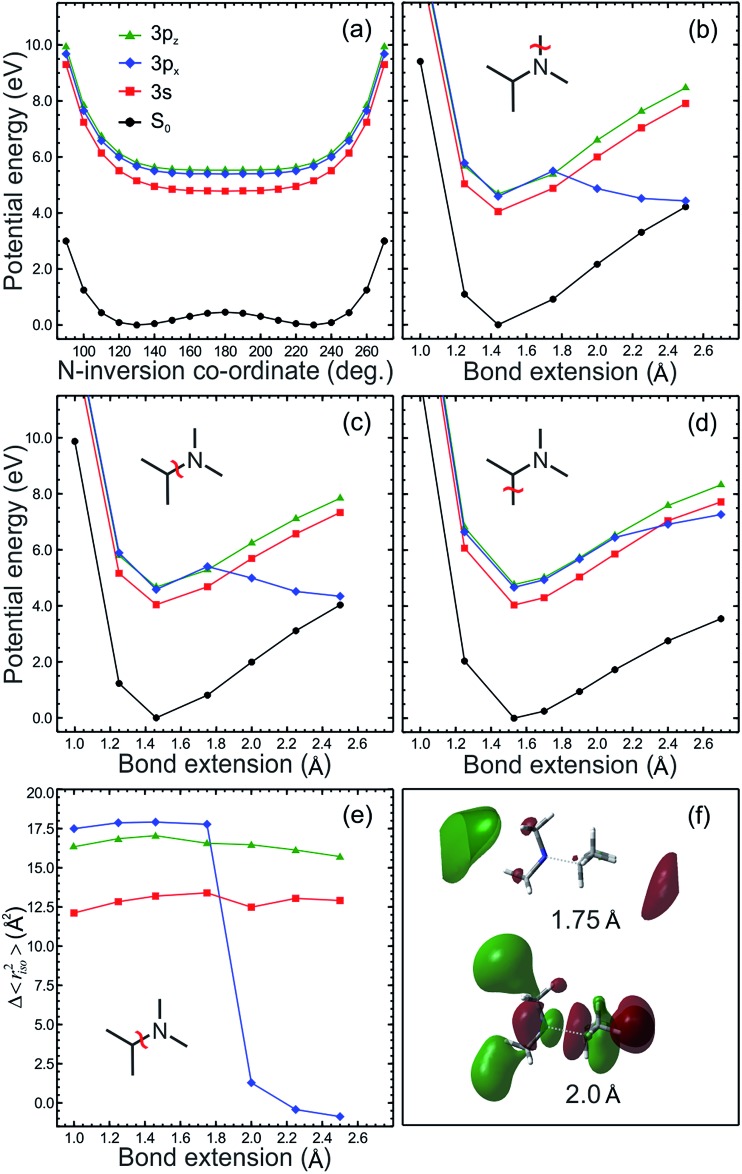
EOM-CCSD/aug-cc-pVDZ potential energy cuts for DMIPA along (a) the nitrogen inversion (or “planarization”) coordinate, (b) the *N*-methyl bond, (c) the *N*-isopropyl bond, and (d) the *C*-methyl bond (corresponding to α-cleavage). The relaxed excited state structure “Con 1” (see Fig. 8) was used as the starting reference geometry. Panel (e) shows the isotropic invariant of the excited-state second-moment of the electronic charge distribution with respect to the ground state along the *N*-isopropyl bond and (f) depicts the largest orbital transition in the EOM-CCSD eigenvectors at 1.75 and 2.0 Å, once again along the *N*-isopropyl bond.

To investigate the possibility of dissociative excited state relaxation channels, EOM-CCSD/aug-cc-pVDZ calculations were performed on DMIPA to obtain potential energy curves along the nitrogen-methyl bond, the nitrogen-isopropyl (N–C_iso_) bond, the carbon-methyl bond (corresponding to α-cleavage) and the nitrogen inversion co-ordinate. These scans were undertaken for relaxed geometries (*i.e.* those planar about the N atom), obtained from a UCAM-B3LYP/6-311G(d,p) constrained series of optimisations on the cation. The output obtained is summarized in Fig. 9(a)–(d). The 3s state remains strongly bound along all coordinates, however, one member of the 3p manifold (3p_*x*_ in this instance) develops increased valence character, and crosses the 3s state at relatively modest bond extensions. As discussed in the Introduction, it has been generally established that states of s Rydberg character evolve significant σ* valence character at even relatively modest N–H and/or N–CH_3_ bond extensions in a wide range of small molecular systems, creating potential pathways for dissociation and/or non-adiabatic decay processes.^9^ It is therefore perhaps surprising that in these TAA species it appears to be Rydberg/valence evolution by a member of the p manifold that facilitates the non-adiabatic dynamics instead. Repeating the calculation using the ground state geometry also produces the same overall behaviour, strongly implying that planarization about the nitrogen atom is not an appreciable factor. Interestingly, we also note that preliminary calculations in *N*-methylisopropylamine (*i.e.* replacing one of the methyl groups in DMIPA with an H atom) suggest that it is now the 3s state that evolves valence character along the N–H stretching coordinate – as would be predicted on the basis of extensive previous work on dynamics mediated by mixed Rydberg/valence states.^9^,^11^ More detailed investigation of these observations is beyond the scope of this current work, but is a clear avenue of great potential photochemical interest to be explored in the near future. Our findings also correlate with the striking differences in relaxation timescales seen between TAAs and their primary and secondary analogues following UV absorption. In the latter cases no long-time dynamics are observed, with overall excited state lifetimes of <200 fs.^12^,^17^

The increase in valence character of the 3p_*x*_ state at extended N–C_iso_ bond distances is also strongly indicated in the evolution of the second moment of the charge distribution relative to the ground state Δ*r*_iso_^2^, as shown in Fig. 9(e). At ∼1.8 Å the 3p_*x*_ state dramatically changes character from Rydberg to valence, as indicated by the sudden drop in the Δ*r*_iso_^2^ value. We note that this crossover is well to the left of the Coulson–Fischer point, and also that the coupled cluster T1 diagnostic^49^,^50^ confirms that a CCSD approach provides a well-balanced reference wavefunction out beyond 2.5 Å. Evolution of increased valence character within the 3p_*x*_ state of DMIPA is yet further illustrated in Fig. 9(f), which contrasts the largest orbital transition in the EOM-CCSD eigenvectors at 1.75 and 2.0 Å along the N–C_iso_ coordinate. At 1.75 Å, the 3p_*x*_ ← n_N_ Rydberg transition dominates (orbital coefficient –0.601). At 2.0 Å, however, the dominant transition is of predominantly σ* ← n_N_ character (coefficient 0.449), with the next largest components being of mixed Rydberg and valence type.

Finally, Fig. 9 also reveals that, although dissociative, the α-cleavage coordinate is much more strongly bound than along either of the N–C bonds. This appears to confirm previously reported observations asserting mass fragments corresponding to α-cleavage in DMIPA following excitation at ∼6 eV arise from fragmentation of the parent ion rather than the excited neutral.^13^

### D. 3p/3s coupling

**Fig. 10:**
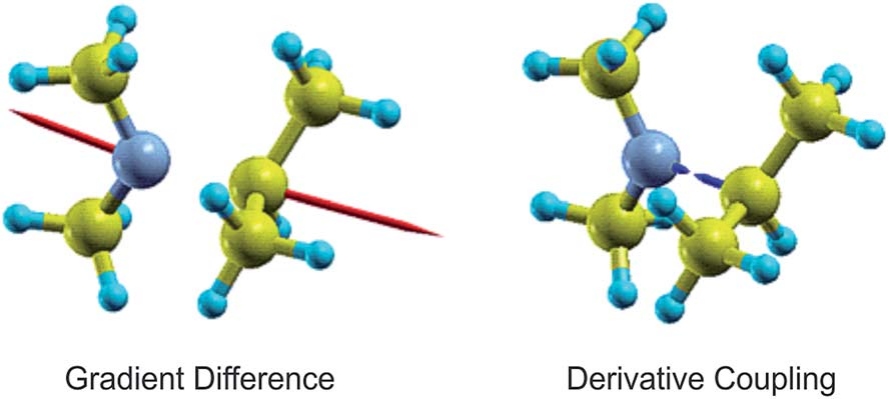
Branching space vectors for the 3s/3p conical intersection (CI) as obtained from CASSCF calculations performed on DMIPA (described in the main text). The derivative coupling and gradient difference vectors define the directions in which the degeneracy is lifted when moving away from the CI point.

To investigate the interaction between the 3s and 3p states, complete active space self-consistent field (CASSCF) calculations were undertaken (in the aug-cc-pVDZ basis), once again for DMIPA. An active space was constructed using 8 electrons in 6 orbitals. This was based upon a mapping of configuration interaction singles (CIS) states to those obtained using TD-CAM-B3LYP and then using the corresponding CIS natural orbitals to generate a wavefunction that gave a balanced description of the ground and four excited states required. Using this CASSCF wavefunction a conical intersection was optimised that connects the 3s and 3p_*x*_ Rydberg states. Branching space vectors (*i.e.*, those defining the plane in which the degeneracy is lifted to first order) are shown in Fig. 10. These vectors are non-orthogonal and have an angle of 157°, with the predominant nuclear motions being the N–C_iso_ stretch and (to a lesser extent) torsion between the two terminal groups. The nature of the branching space is somewhat reminiscent of the S_1_/S_2_ conical intersection in thioanisole.^51^ Attempts to locate conical intersections within the 3p manifold proved unsuccessful as the states were too heavily mixed to permit any meaningful analysis. A separate search for a conical intersection between the 3s and S_0_ states revealed no evidence of any non-adiabatic interaction. We note that this finding also appears to be confirmed by the experimental PAD data, as will be expanded upon further in Section V-B.

In addition to the non-adiabatic coupling between the 3p_*x*_ and 3s states in DMIPA, we have also considered the diabatic nature of these states across the low-lying potential energy surfaces. The aim here is to understand how much mixing there is in each state between the pure “diabatic” s and p Rydberg components. We quantify this as the ratio of the appropriate diffuse p-type basis functions to s-type basis functions in each state, weighted by the associated orbital transition coefficients in the EOM-CCSD eigenvectors. We have analysed three specific geometries: the S_0_ minimum, the fully relaxed excited state minimum (“Con 1” in Fig. 8) and the 3s/3p_*x*_ conical intersection discussed above. For these we observe, respectively, that this measure for the 3s state goes as 0.488 → 0.269 → 0.465, and for the 3p_*x*_ state as 1.295 → 2.155 → 1.604. Thus, at the relaxed minimum energy geometry the 3s state exhibits “purest” s-character (as the p/s ratio is smallest) and the 3p_*x*_ state the “purest” p-character (as its p/s ratio is largest) relative to the other geometries considered.

## V. Discussion

Photoelectron spectra presented in Fig. 5 for all three systems look broadly similar in many respects to those reported previously for a number of other TAA systems.^22^,^23^,^25^ This body of earlier work, which we have already drawn upon in previous sections, provides a starting point for discussing the dynamics seen in our data: the photoelectron peak appearing at lower Rydberg binding energy (∼2.3 eV) is attributable to ionization from the initially prepared 3p manifold. Internal conversion then occurs on a sub-picosecond timescale to the 3s state giving rise to the delayed onset photoelectron peak at higher Rydberg binding energies (∼3.0 eV). The 3s state then decays on a much more extended timescale. Both of the observed peaks in the photoelectron spectra are narrow (indicating a strong propensity for diagonal ionization) and exhibit a high degree of angular anisotropy (suggesting well-defined orbital angular momenta). These observations reflect the predominant Rydberg character of the excited states overall. We now explore the more subtle dynamical features revealed by our data in more detail.

### A. Short-time (<1 ps) dynamics

The shapes and relative amplitudes of both the photoelectron spectra (Fig. 5) and associated DAS plots (Fig. 6) indicate that DMPA and DMIPA appear to undergo similar dynamical evolution within the 3p manifold. This is evidenced by the clear spectral shift in the 3p ionization feature (∼2.3 eV). Conversely, in Mpyr this shift is associated with the 3s ionization feature (∼3.0 eV). Similar short-time evolution of photoelectron signals have been reported previously in several other TAA systems, being attributed to internal conversion within the 3p manifold^22^ and, in larger (less rigid) species, to additional conformational relaxation effects.^23^–^25^ Based on the calculations presented in Section IV, there are a number of relaxed conformers that can be accessed on the excited state potential energy surfaces (see Fig. 8). Calculations also reveal (see Table 1) that in Mpyr the transition to the lowest-lying member of the singlet 3p manifold carries the highest oscillator strength (3p_*y*_ ← n_N_). As such, no significant internal conversion *within* the 3p manifold is likely to be observed in Mpyr. The initially prepared 3p_*y*_ population therefore rapidly and directly decays to the 3s state (as described by *τ*_1_ = 400 ± 30 fs) where conformational reorganization then gives rise to the small observed spectral shift (as described by *τ*_2_ = 640 ± 50 fs). The link between the spectral shift and the conformational dynamics may be rationalized in terms of the small changes in the perturbation the outgoing electron “feels” from the (non-spherical) molecular ion core – effectively inducing changes in the quantum defect of the Rydberg state as the nuclear geometry evolves.

In DMIPA and DMPA the situation is different. Here it is the transition to the state sitting energetically in the middle of the 3p manifold that carries the largest oscillator strength. Following 200 nm excitation internal conversion within the 3p manifold may also now form a significant element of the overall relaxation prior to decay into the 3s state. Given the ∼0.1 eV splitting between the various 3p states (see Table 1), internal conversion alone could account for the small spectral shift seen in the photoelectron peak at ∼2.3 eV. However, the excited state population also now resides in the 3p manifold for a longer period of time than in Mpyr (the overall 3p residence times are 560 ± 40 fs and 640 ± 40 fs for DMIPA and DMPA, respectively – see Section III-C. This is significantly longer than *τ*_1_ = 400 ± 30 fs for Mpyr). Conformational reorganization is also therefore able to take place and contribute to the observed spectral shift before crossing to the 3s state takes place. In DMIPA and DMPA internal conversion within the 3p manifold and conformational reorganization therefore contribute the spectral shift seen in the peak at ∼2.3 eV (as described by *τ*_1_) – before decay to the 3s state (described by *τ*_2_). Consequently, no evolution of the central peak position is then seen at ∼3.0 eV in DMIPA and DMPA. This interpretation of the data appears fully consistent with similar observations reported in other TAA systems as well as the predictions provided by our own calculations. Additionally, since geometry relaxation occurs before internal conversion in DMIPA and DMPA but afterwards in Mpyr we may infer that planarization about the N atom (the major conformational change taking place) is not a significant prerequisite for efficient internal conversion to the 3s state. This observation also appears to be in excellent agreement with the calculated branching space vectors for DMIPA presented in Fig. 10.

At a purely “atomic” level of interpretation, single-photon ionization from a Rydberg state of exclusively p character should give rise to only s and d photoelectron partial waves on the basis of angular momentum conservation (Δ*l* = ±1). The shape of the resulting PAD is not only dependent on the amplitudes of these partial wave components but also their relative phases. PADs associated with ionization from the 3p manifold in our data are directed predominantly along the direction of the laser polarization (see Fig. 3), suggesting a relatively small (<30°) phase shift between the s and d partial waves.^4^ It has been previously argued^52^ that the scattering phase *η*_*l*_ may be approximated using the quantum defects *δ*_*l*_ of Rydberg states in the limit of high principal quantum numbers *via* the relationship *η*_*l*_ = π*δ*_*l*_.^53^ Although no high-*n* quantum defect data exists for the three systems considered here, experimentally determined values have been reported for both NH_3_ and the caged TAA system DABCO.^54^,^55^ On the basis of this data, quantum defects in the region of 0.9–1.1 and 0.0–0.1 might reasonably be assumed for the s and d Rydberg series, respectively. However, this range of values suggests a relative *η*_s_–*η*_d_ phase shift of >140° (*i.e.* PADs associated with 3p ionization should peak *perpendicular* to the laser polarization). This discrepancy may be a consequence of two principal factors: (i) mixed orbital angular momentum electronic character of the “p” state and (ii) the possibility of the outgoing electron wavefunction scattering off the non-spherically symmetric ion core (*i.e.* a failure of the “atomic” picture assumed as a starting point for discussion) – this must clearly be significant in light of the spectral shifts seen due to conformational rearrangement in the photoelectron spectra in Fig. 5. In the absence of a rigorous theoretical evaluation (beyond the scope of this present work) we are unable to comment further, although our observations suggest that TAA systems may provide interesting and convenient experimental benchmarks for future theoretical work investigating molecular PADs and the associated scattering dynamics in more detail.

Once again starting from an “atomic” viewpoint, single-photon ionization from the 3s state should give rise to photoelectron partial waves of predominantly p character, leading to PADs peaking strongly along the laser polarization direction. This is indeed observed, although the β_2_ values obtained from our data fits are less than the limiting value of 2.0, possibly again reflecting the somewhat mixed character of the state. Additionally, since pure s states cannot possess any inherent angular momentum alignment, the β_4_ component associated with ionization from such an orbital should be zero. This is clearly not reflected in the PAD data for the 3s ionization feature over the first 500 fs of pump–probe delay (see Fig. 7) – although at longer delay times there is then an extended time period where β_4_ then is equal to (or very close to) zero before a subsequent long-time rise (considered later) is then seen. This observation appears consistent with the orbital composition calculation discussed in Section IV, where the character of the 3s state was shown to be “purer” at the relaxed excited state geometry than at the S_0_ geometry.

Overall, the rapid dynamical evolution of both β_2_ and β_4_ seen at short (<1 ps) times for ionization of the 3s and 3p states provides a signature of the non-adiabatic coupling process leading to internal conversion from the 3p manifold. A more detailed analysis/discussion of the data is, however, challenging due to the fact that additional dynamical factors also influence/convolute the observed form of the PADs. Primarily these are (i) changes in the relative phases and amplitudes of the outgoing photoelectron partial waves – a consequence of conformational rearrangement modifying the scattering dynamics off the non-spherical ion core potential and (ii) significant changes in excited state Rydberg *vs.* valance character as a function of nuclear coordinates. Any loss of “Rydbergization”^56^ is a different effect to changes in electronic character induced by state mixing interactions – as has been considered in some detail for the case of H_2_O.^45^ In DMIPA and DMPA additional evolution in the short-time PAD data may also arise due to internal conversion within the 3p manifold although, given the similarity of the data in Fig. 7 for all three systems, we assume this is a minor factor.

### B. Long-time (>1 ps) dynamics

All three TAA systems exhibit relatively long decay times for the 3s state following population *via* the 3p manifold. In DMIPA, however, the 3s lifetime is noticeably shorter (*τ*_3_ = 38 ps) than for DMPA (*τ*_3_ = 130 ps) and Mpyr (*τ*_3_ = 160 ps). This observation may be rationalized using the energy level data presented in Table 1: since the 3s state in DMIPA is predicted to sit ∼0.25 eV lower than in DMPA and MPyr, it will therefore possess a higher level of associated vibrational excitation following internal conversion. Weber and co-workers have previously demonstrated an exponential decrease in DMIPA 3s lifetime with increasing excess vibrational energy.^35^ More generally, the exponential dependency of both radiative and non-radiative decay rates on excited state internal energy is also a typically expected outcome. As such, we may assume that the higher quantity of excess energy deposited into the vibrational coordinates of DMPIA (relative to DMPA and Mpyr) is primarily responsible for the reduced 3s state lifetime. This assertion does not, however, say anything about the physical nature of the decay mechanism and therefore reveal the ultimate fate of the 3s state. As discussed previously, radiative decay *via* fluorescence has been suggested as a possible pathway,^13^ although it seems unlikely this is the dominant relaxation mechanism (following 200 nm excitation) on the basis of quantum yield data reported for several other TAA systems.^14^–^16^ It is also difficult to see how fluorescence decay leads to increased β_2_ and β_4_ values at long pump–probe delay times (see Fig. 7) as this process does not directly couple the initial and final electronic states. In this long-time limit, the anisotropy evolution must primarily be a consequence of a coupling interaction as the system is fully relaxed and major conformational rearrangements – which may also influence the PAD evolution at short times – are now complete.

A second option for 3s decay is that of intersystem crossing (ISC), although we believe this may also be ruled out for several reasons: (i) predicted triplet state energies are only ∼0.05–0.15 eV below the corresponding singlet states (see Table 2). Given the high (10.85 eV) total pump + probe photon energy relative to the *D*_0_ ionization potentials of all systems (8.20–8.41 eV)^57^–^59^ our TRPEI experiments therefore allow us to project very deeply (2.45–2.65 eV) into the ionization continuum (see Fig. 4). Therefore even in the event of significant triplet state geometry changes leading to very different ionization Franck–Condon factors relative to the singlet states, we would not reasonably expect to be “blind” to triplet state population – *i.e.* efficient triplet state ionization should be possible in our experiments but we see no signatures of this process in the experimental data, (ii) since the lowest component of the triplet 3p manifold sits >0.45 eV above the singlet 3s state in all three TAA systems (once again, see Table 2), efficient ISC between these levels is likely to be disfavoured of the basis of simple density-of-states arguments, (iii) given the previous point, this only leaves the triplet 3s state as a potential ISC pathway. This process (between states of the same orbital character) would be strongly disfavoured by El-Sayed's rules and therefore unlikely to occur to any significant extent in ∼100 ps.^60^ Coupling between two electronic states of predominantly s orbital character would also be unlikely to produce the significant non-zero β_4_ values seen in Fig. 7 at extended pump–probe delays.

Finally, although our computational attempts to locate a 3s/S_0_ conical intersection proved unsuccessful (see Section IV), a third possibility to consider for the fate of the 3s state is that of direct non-radiative relaxation back to S_0_. However, such a process appears to be ruled out by the data in Fig. 7 as prerequisite mixing of the overwhelmingly valence S_0_ character into the 3s state would be extremely unlikely to produce *increased* β_2_ values (characteristic of more well-defined angular momentum) at long pump–probe delays (in fact, a significant decrease would be predicted).

Having eliminated the above possibilities, we therefore arrive at the following conclusion for the ultimate fate of the 3s state in all three systems following 200 nm excitation: once initially prepared *via* ultrafast internal conversion from the 3p manifold, population spends an extended time (tens of picoseconds) on the 3s surface. During this period, the initially localized vibrational wavepacket is dispersed and intramolecular vibrational redistribution (IVR) will also occur. Consequently, the system may explore a large volume of phase space, occasionally re-encountering the conical intersection with the 3p state (the specific manifold component label – *x*, *y*, or *z* – will vary between systems). At this time, re-crossing to the dissociative part of the 3p surface may take place, leading to either rapid direct bond fission along an N–C stretching coordinate or (potentially) subsequent non-adiabatic decay to S_0_*via* an additional conical intersection at highly extended N–C bond lengths (see Fig. 9) – both processes to which our current TRPEI measurements are “blind” as the population has then exited the ionization detection window (and so we are unable to comment further on this aspect of the dynamics at present). Close to the 3s/3p conical intersection geometry, increased state mixing (as suggested by our calculations) gives rise to a component within the overall ionization signal from the 3s state exhibiting increased β_2_ and β_4_ values. As IVR proceeds, this effect gains in significance, leading to the observed evolution of the anisotropy at large pump–probe delay times. This mechanism also perhaps explains why population in the 3s state only starts to decay *via* encounters with the 3s/3p conical intersection at extended delay times, as energy must be redistributed into specific vibrational modes to facilitate efficient access to the repulsive part of the 3p potential. On the basis of our DMIPA calculations, likely candidates are N–C stretching modes. Overall, this dynamical interpretation appears to fit with the experimental and theoretical evidence currently available although we stress the need for further complementary studies to strengthen our initial conclusions.

## VI. Summary

Time-resolved photoelectron imaging was used to investigate non-adiabatic relaxation dynamics operating in a series of three tertiary aliphatic amines following excitation at 200 nm. This was supported by *ab initio* calculations investigating several molecular properties at various levels of theory. Sub-picosecond internal conversion between the initially excited 3p Rydberg manifold and the lower-lying 3s state was observed to be convoluted with additional dynamical process involving internal conversion within the 3p manifold and conformational reorganization of the molecular framework. Perhaps surprisingly, after an extended period on the 3s surface all three systems then appear to undergo a re-crossing back to one of the 3p states before rapidly exiting the detection window of the experiment (presumably either by dissociating along one of the N–C stretching coordinates or non-adiabatically populating S_0_). Overall, the non-adiabatic dynamics appears to be primarily mediated by the evolution of valence character as N–C bonds are extended within a member of the 3p Rydberg manifold. This is somewhat different behaviour to that seen in primary and secondary aliphatic amines (as well as in aromatic systems such as aniline) where evolution of valence character within the 3s state is the key diver of ultrafast population transfer. Our findings offer a clear demonstration of the detailed insight afforded by the energy- and angle-resolved TRPEI methodology. However, the subtle new aspects of the UV relaxation dynamics revealed in TAA systems also highlight the need for additional experimental and theoretical investigations (*e.g.* use of vacuum ultraviolet probes to expand the “view” along the reaction coordinate, observing the time-resolved appearance of photoproducts, performing excited state wavepacket trajectory calculations, *etc.*). This is not only required to further understand our initial observations, but also definitively establish the extent to which they apply more generally in other systems. We anticipate that our current work will serve as a stimulus for such undertakings.
